# Draft Genome Sequence of *Francisella noatunensis* subsp. *orientalis* STIR-GUS-F2f7, a Highly Virulent Strain Recovered from Diseased Red Nile Tilapia Farmed in Europe

**DOI:** 10.1128/genomeA.01555-16

**Published:** 2017-03-16

**Authors:** José Gustavo Ramírez-Paredes, Pär Larsson, Stefanie Wehner, Michaël Bekaert, Caroline Öhrman, Matthijs Metselaar, Kimberly Dawn Thompson, Randolph Harvey Richards, David James Penman, Alexandra Adams

**Affiliations:** a Institute of Aquaculture, Faculty of Natural Sciences, University of Stirling, Stirling, United Kingdom; b Swedish Defence Research Agency (FOI), CBRN Defence and Security, Umeå, Sweden; c The Fish Vet Group, Inverness, United Kingdom; d Aquaculture Research Group, Moredun Research Institute, Edinburgh, United Kingdom

## Abstract

A highly virulent strain of *Francisella noatunensis* subsp. *orientalis*, STIR-GUS-F2f7, was isolated from moribund red Nile tilapia (*Oreochromis niloticus*) farmed in Europe. In this communication, the complete genome sequencing of this bacterium is reported.

## GENOME ANNOUNCEMENT

*Francisella noatunensis* subsp. *orientalis* is a Gram-negative, nonmotile, nonsporulating, aerobic, intracellular, fastidious, and pleomorphic coccobacillus (size, 0.2 to 1.7 µm) associated with systematic granulomatous disease in tropical farmed and ornamental fish, for which currently no prophylactic treatment exists (1). In November 2012, a highly virulent strain of* F. noatunensis* subsp. *orientalis*, STIR-GUS-F2f7, was recovered from diseased red Nile tilapia (~10 g/5.5 cm) farmed in a recirculating system in Europe, where mortality rates were around 60% (2).

The genomic DNA of STIR-GUS-F2f7 was extracted as outlined by Seward et al. (3). The purity and concentration of the DNA were assessed by electrophoresis using a 1% agarose gel stained with ethidium bromide at 0.001%, and 260/280-to-260/230 ratios were determined using a NanoDrop ND1000 (Thermo Scientific, DE). The final DNA concentration was adjusted to 2 µg and assessed with a Qubit 2.0 fluorometer (Invitrogen Life Technologies, Inc., CA). Libraries were prepared from 1 µg of DNA with the TruSeq 100-bp cycle paired-end DNA sample prep kit, and the genome was sequenced using the HiSeq 2500 platform (Illumina, CA, USA) with version 3 sequence chemistry. The parallel paired-end sequence assembler ABySS version 1.3.5 (4) was used for *de novo* assembly, which resulted in a total of 10 contigs, all of which had a length longer than 200 bp, an *N*_50_ of 482,249 bp, and a coverage of 11×. The genome of STIR-GUS-F2f7 consists of 1,887,094 bp, with no plasmids and a G+C content of 32.4%. RNAmmer version 1.2 (5) predicted 19 copies of rRNA genes: seven 5S and six of 16S and 23S. The genome annotation performed with the NCBI Prokaryotic Genome Annotation Pipeline version 3.1 (6) predicted a total of 1,892 genes, of which 1,451 were protein-coding sequences, 45 were tRNA, four were noncoding RNAs (ncRNA), and 373 were pseudogenes. Of the protein-coding genes, the RAST server (7) predicted 239 as being involved in protein metabolism; 199 in the synthesis of amino acids and derivatives; 122 in carbohydrate catabolism; 116 in cofactors, vitamins, prosthetic groups, or pigment production; 100 in RNA metabolism; 95 in fatty acid, lipid, and isoprenoid synthesis; 82 in DNA metabolism; 79 in respiration; 74 in capsule and cell wall synthesis; 71 in stress response; 59 in nucleoside and nucleotide synthesis; 54 in membrane transport; 32 in virulence, disease, and defense mechanisms; 24 in cell division and cell cycle; 17 in potassium metabolism; 16 in regulation and cell signaling; 11 in phosphorus metabolism; 10 in iron acquisition and metabolism; 10 in sulfur metabolism; six in the metabolism of aromatic compounds; five in secondary metabolism; five in nitrogen metabolism; one in dormancy; and 34 as miscellaneous proteins.

At present, no *F. noatunensis* subsp. *orientalis* genomes from Europe are available in public databases. The genome sequence of STIR-GUS-F2f7 will facilitate comparative analyses with less virulent *F. noatunensis* subsp. *orientalis* strains from different geographical origins.

### Accession number(s).

This whole-genome assembly has been deposited at DDBJ/EMBL/GenBank databases under the BioProject PRJNA297804. The genome accession number is LTDO00000000, and the version described in this paper is LTDO01000000. The raw sequences have been submitted to the Sequence Read Archive (SRA) database under the accession number SRP080830.
